# Genome-wide transcriptional analysis of submerged lotus reveals cooperative regulation and gene responses

**DOI:** 10.1038/s41598-018-27530-x

**Published:** 2018-06-15

**Authors:** Bei Wang, Qijiang Jin, Xiao Zhang, Neil S. Mattson, Huihui Ren, Jing Cao, Yanjie Wang, Dongrui Yao, Yingchun Xu

**Affiliations:** 1 College of Horticulture, Nanjing Agricultural University, Nanjing, 210095 China; 2 Horticulture Section, School of Integrative Plant Science, Cornell University, Ithaca, USA; 3 Institute of Botany, Jiangsu Province and Chinese Academy of Sciences, Nanjing, 210014 China

## Abstract

Flooding severely limits plant growth even for some aquatic plants. Although much work has been done on submergence response of some important crop plants, little is known about the response mechanism of aquatic plants, i.e. lotus (*Nelumbo nucifera*). In this study, we investigated the genome-wide regulation lotus genes in response to submergence stress by high-throughput mRNA sequencing. A total of 4002 differentially expressed genes (DEGs) in lotus upon submergence stress. Among them, 1976 genes were up-regulated and 2026 down-regulated. Functional annotation of these genes by Gene ontology (GO) and Kyoto encyclopedia of genes and genomes (KEGG) enrichment analysis revealed that they were mainly involved in processes of oxidation-reduction, abiotic stimuli, cellular metabolism and small molecule metabolism. Based on these data, previous work and quantitative RT-PCR (RT-qPCR) validation, we constructed a cooperative regulation network involved in several important DEGs in regards to the antioxidant system, disease resistance, hypoxia resistance and morphological adaptation. Further work confirmed that several innate immunity genes were induced during submergence and might confer higher resistance to lotus rot disease. In conclusion, these results provide useful information on molecular mechanisms underlying lotus responses to submergence stress.

## Introduction

Flooding, one of the most common stresses is known to affect plant growth, development and distribution. Many important terrestrial crop plants, i.e. cotton, corn, rice and even aquatic plants (such as lotus), are often subjected to heavy rain or irrigation-caused submergence. Flooding and submergence conditions can result in severe yield losses in many crop species^1^.

Complete submergence causes a much more stressful scenario for plants as all organs are covered by water and shoots suffer from shortages of carbon and oxygen^2^. Meanwhile, the energy supply of plants becomes insufficient because aerobic respiration and photosynthesis are largely inhibited during complete submergence. Submergence also results in a restriction in light availability^3^, which influences cellular energy and carbohydrate shortages of plants. Moreover, re-oxygenation of the tissues and organs can also result in cellular damage from reactive oxygen species (ROS)^4^.

Physiological and molecular processes underlying the response to submergence in plants have been well studied. To cope with oxygen loss, some waterlogging-tolerant plants have developed metabolic and morphological adaptations which can enable them to survive transient periods of complete or partial submergence^5^. In order to survive an energy and carbohydrate shortage caused by submergence stress, plants can regulate consumption of energy reserves and carbohydrates or develop morphological adaptations. For instance, under anoxia, rice and some other plants are able to elongate coleoptiles and stems. Meanwhile, oxidative respiration is shut-down and anaerobic respiration is enhanced because of low-oxygen status^6^.

High-throughput sequencing (HTS) technology also known as “Next-generation sequencing technology” has become a widely used method in analyzing transcriptomes qualitatively and quantitatively^7^. Genome-wide analysis and expression studies in model plants, such as *Arabidopsis thaliana*, show rapid and specific changes in transcriptional and translational levels, affecting 5–10% of the transcriptome^8–12^. Transcription factors, such as WRKY22 and AP2/ERF transcription factors in Arabidopsis and Sub1A^13,14^, SNORKEL1 and 2 in rice^15^, have been found to regulate gene expression under hypoxia stress. In the WRKY genes tested, WRKY22 had the highest induction level at the early stages of flooding. At the same time, AP2/ERF is also one of the most important gene families that are involved in plant response to biotic and abiotic stresses^14^. In some submergence-tolerant rice varieties, they could maintain viability by limiting underwater elongation under submergence stress. Acclimation responses to submergence are harmonized by the submergence-inducible Sub1A which encodes an ethylene-responsive factor-type transcription factor (ERF). The expression of genes involved under submergence is mediated by multiple levels of transcriptional, post-transcriptional, translational and post-translational regulation, including phosphorylation and protein degradation. In particular, protein degradation may be involved in the negative regulation of hypoxia response^16^. Moreover, submergence stress also changed the abundance of proteins involved in protein transport and storage, adenosine triphosphate (ATP) synthesis, metabolism, and signal transduction^17^.

Lotus (*Nelumbo Nucifera*) is a well-known and economically important ornamental plant in Asian countries, such as China, Japan, India and Korea^18^. Although lotus is an aquatic plant, it still suffers from submergence stress caused by flooding or heavy rain in the summer^19^. Submergence stress causes severely reduced yield in lotus^20^. Therefore, understanding the mechanisms of submergence stress responses in lotus is important. In this study, high-throughput sequencing was performed on the Illumina platform to examine differential gene expression under waterlogging stress in lotus. The purpose of this study was to identify submergence-responsive genes and further analyze the molecular mechanisms underlying submergence stress response of lotus. RT-qPCR assays were used to confirm the expression pattern of several submergence-responsive genes. These results could expand our understanding of regulatory gene expression profiles underlying submergence response of lotus, and provide useful information to design engineering strategies towards enhanced stress tolerance in lotus and other plants.

## Results and Discussion

### Mapping and Quantitative Assessment of Illumina Sequence Data

Flooding is a frequent natural disaster in many regions of the world, which can inhibit aerobic respiration and photosynthesis, and drastically reduce yield of plants. Although lotus is a well-known aquatic plant, it is also suffers from submergence caused by heavy rain and flooding. To extend our understanding of the molecular mechanisms of lotus in the survival of waterlogging, we performed a comparative transcriptomic analysis of lotus upon complete submergence.

**Figure 1:**
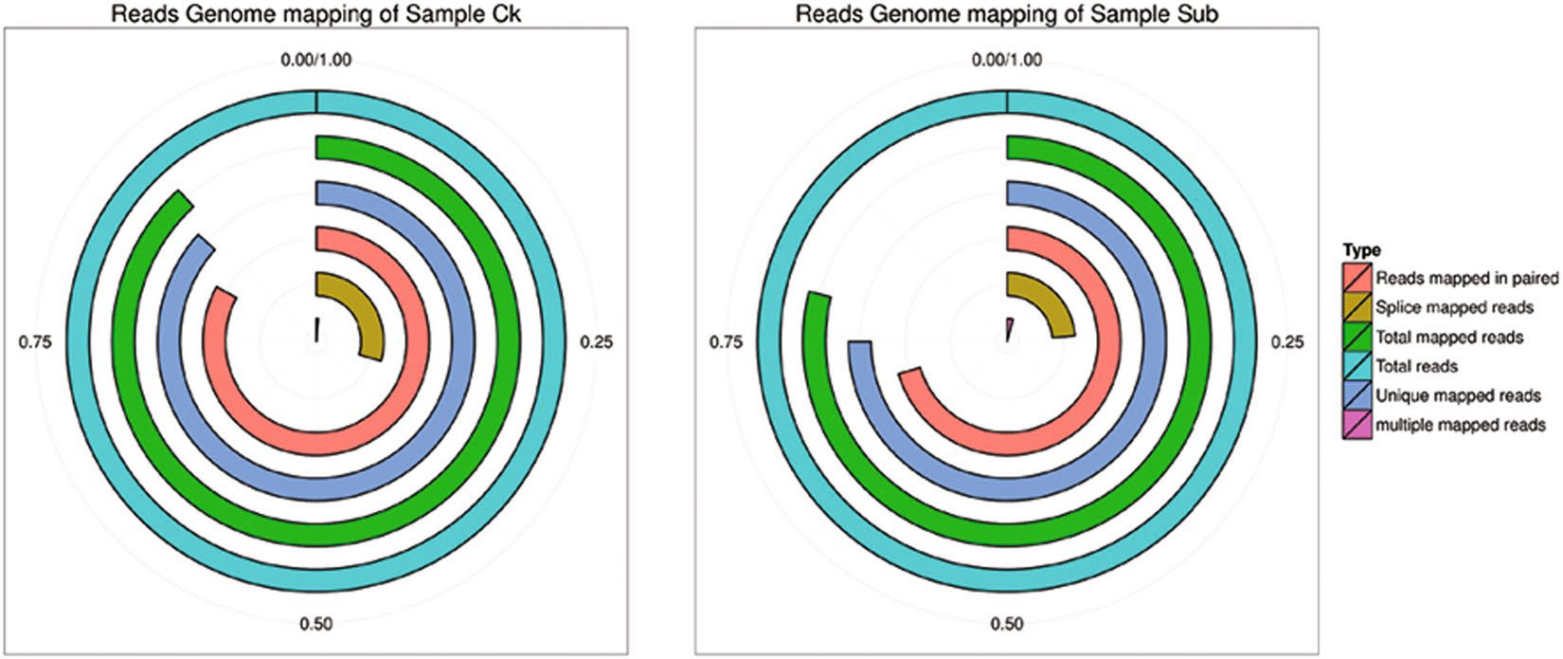
Clean reads in Ck (control) and Sub (submergence treatment) libraries. The raw reads were filtered to generate clean reads which were then mapped to the lotus genome using HISAT2. Unique mapped reads, reads that map to only one location of the reference genome. Each circle represents a match type, the radius of the circle represents the ratio of different match type.

A total of 50.28 M and 50.86 M raw reads were generated from Ck (Control) and Sub (Complete submergence) libraries with Illumina Solexa sequencing technology, respectively (Table 1). After trimming the raw reads to remove adaptor sequence, empty reads, and low-quality sequences, a total of 50.24 M (99.92%) and 50.81 M (99.91%) high-quality reads (clean reads) were generated from raw reads of Ck and Sub libraries, respectively, with a Q20 percentage over 97%, Q30 percentage over 95%, and a GC percentage between 47.2 and 49% (Table 1). The majority of clean reads (78.89 and 88.19% in Ck and Sub libraries, respectively) were successfully mapped to the latest lotus genome (http://www.ncbi.nlm.nih.gov/genome/?term=nelmbo+nucifera) (Fig. 1, Table S1). It was found that 86.66% (Ck) and 75.00% (Sub) of the clean reads mapped to one position in lotus genome, while 82.90% (Ck) and 70.40% (Sub) of the clean reads could be mapped to multiple position.

**Table 1:** Reads from RNA-Seq library sequencing.

Type	Sub	Ck
Total Raw Reads	50851992	50284154
Total Low Quality Reads	7816	7354
Total Low Quality Reads Ratio (%)	0.02	0.01
Total Clean Reads	50805686	50242328
Total Clean Reads Ratio (%)	99.91	99.92
Clean Reads Q20 (%)	97.67	97.53
Clean Reads Q30 (%)	95.75	95.51
Clean Reads GC (%)	48.94	47.24

### Identification and Functional Analysis of DEGs Under submergence Stress

**Figure 2:**
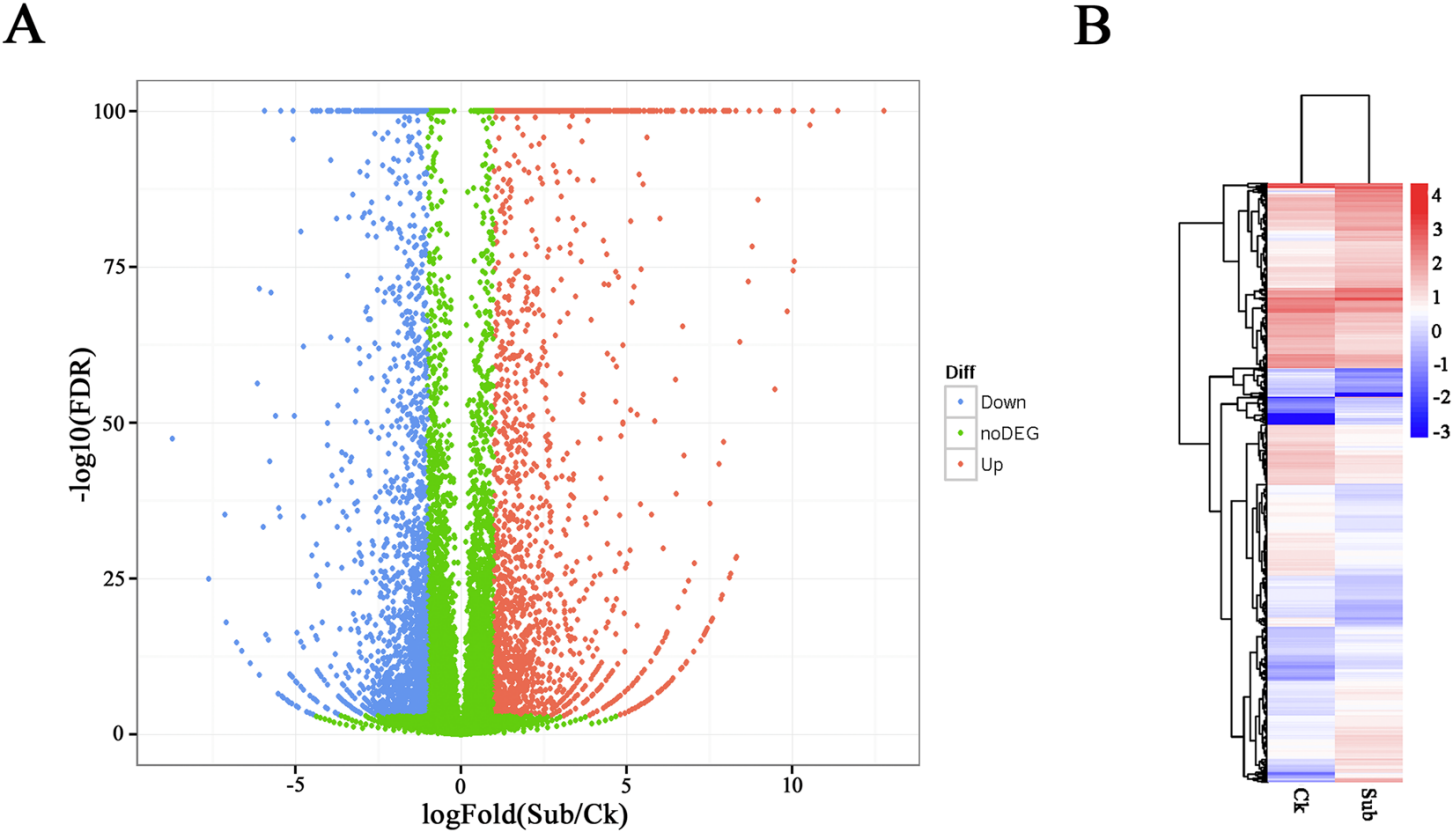
Volcano (**A**) and heatmap plot (**B**) of differentially expressed genes (DEGs). (**A**) Volcano plot visualizing the DEGs between the two different libraries. The p-value < 0.05 was used as a threshold to determine the significance of DEGs. Red dots represent up-regulated genes, blue dots show down-regulated genes, and green dots indicate transcripts that did not change significantly in the Sub (Submergence treatment) compared with Ck (Control). (**B**) Hierarchical clustering of all the DEGs based on log_10_ RPKM values. The X axis represents the two compared samples (Ck and Sub). The Y axis represents DEGs. The color (from blue to red) represents gene expression intensity from low to high.

Distinctive expression patterns of genes between Ck and Sub libraries provides an opportunity to find important genes that were functionally responsive to submergence stress. We conducted a comparative expression analysis of genes between Ck and Sub libraries by a significance-based comparison applying a false discovery rate (FDR)-corrected false positive rate of 0.1% (FDR < 0.001) and a threshold of 2-fold change (log_2_ scale)^21^. In total, 4002 genes were identified to be differentially expressed in lotus upon submergence (Fig. 2A). We used hierarchical clustering of all the DEGs to observe the gene expression patterns, and it was evaluated by log10 per kilobase per million reads (RPKM) for the two groups (Fig. 2B). There was a slight increase in gene frequency, with 50.62% of significantly changed genes experiencing an increase in expression in response to submergence.

**Figure 3:**
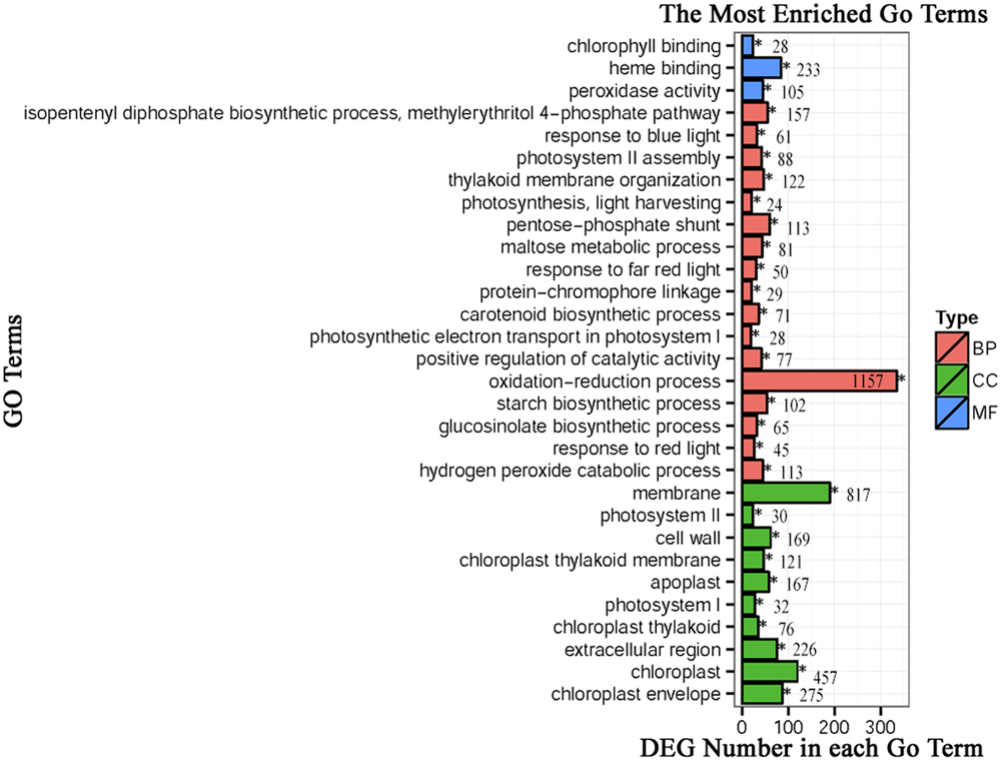
GO classification of differentially expressed genes (DEGs). The most enriched (corrected p-value < 0.001) GO terms of DEGs were summarized. The X axis represents number of DEGs. The Y axis represents GO terms. Red, green and blue represent DEGs enriched under biological process (BP), cellular component (CC) and molecular function (MF) categories, respectively. The number after the GO term is the total number of genes in the corresponding term.

The GO consortium provides a standardized and hierarchical vocabulary (GO terms) to describe the function of gene products and classifies genes into functional categories. Of the 4002 significantly changed genes, 30 significantly enriched GO terms were obtained, divided into three main categories (biological processes, cellular components and molecular functions). The most enriched GO terms (p value < 0.001) are presented in Fig. 3. The most highly represented biological GO terms was ‘oxidation-reduction process’ (GO: 0055114). Under complete submergence, mitochondrial respiration is impaired under anoxia which can lead to the accumulation of reactive oxygen species (ROS) such as superoxide and hydrogen peroxide. Overproduction of ROS results in oxidative stress, causing lipid peroxidation and cell damage^22,23^. Thus, plant cells utilize efficient strategies to reestablish the homeostasis of ROS through modulating genes involved in oxidation-reduction processes^24^, which is consistent with our observation from GO annotation. The cellular component classification most enriched were the ‘membrane’ (GO: 0016020). In terms of molecular function, ‘heme binding’ (GO: 0020037) represented the most abundant subcategories.

**Figure 4:**
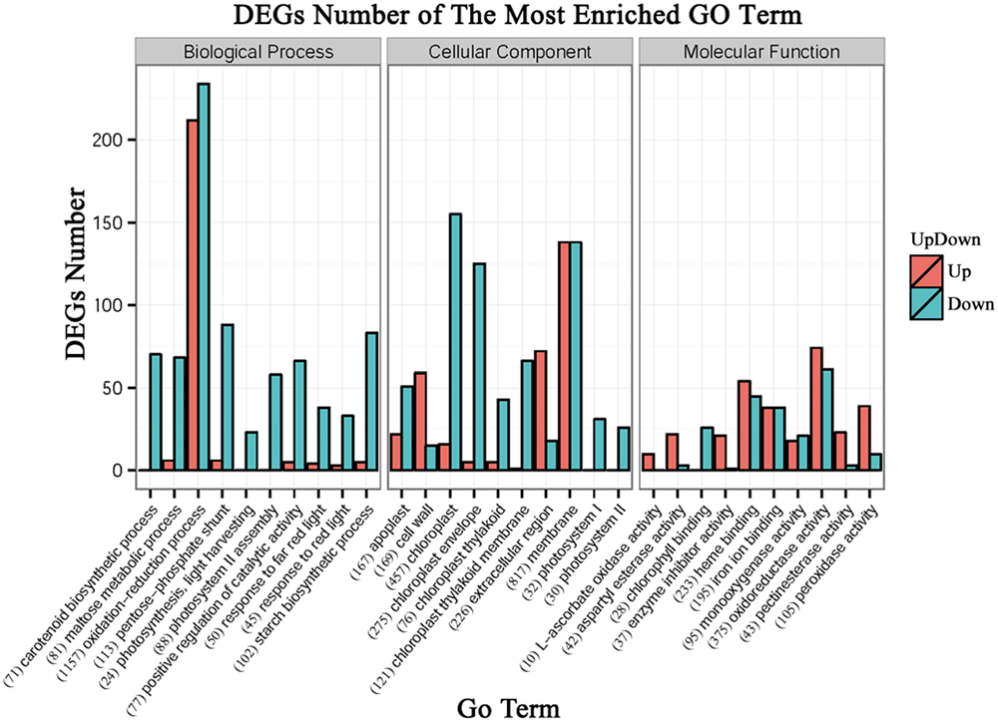
Functional classification of assembled unigenes based on gene ontology categorization. Up = enriched GO term of submergence-upregulated genes; Down = enriched GO term of submergence-downregulated genes; The number in brackets indicates the total number of genes in the GO term. The number after the GO term is the total number of genes in the corresponding term.

Based on the categorization of up-regulated and down-regulated genes respectively, we performed a comparison between these categories and find that there were more down-regulated genes than up-regulated ones in most categories (Fig. 4). In rice, it is also observed that more genes were down-regulated under flooding condition^25^. Interesting, most of genes that involved in oxidation-reduction process were down-regulated. Besides some negative regulators of antioxidant processes, there may be relatively few genes were involved in maintaining ROS homeostasis under complete submergence, which need further investigation. It is worth noting that most photosynthesis-related genes, i.e. Go term: “photosynthesis”, “photosystem II assembly”, and “response to red light”, were downregulated, which could reduce unnecessary energy consumption.

**Figure 5:**
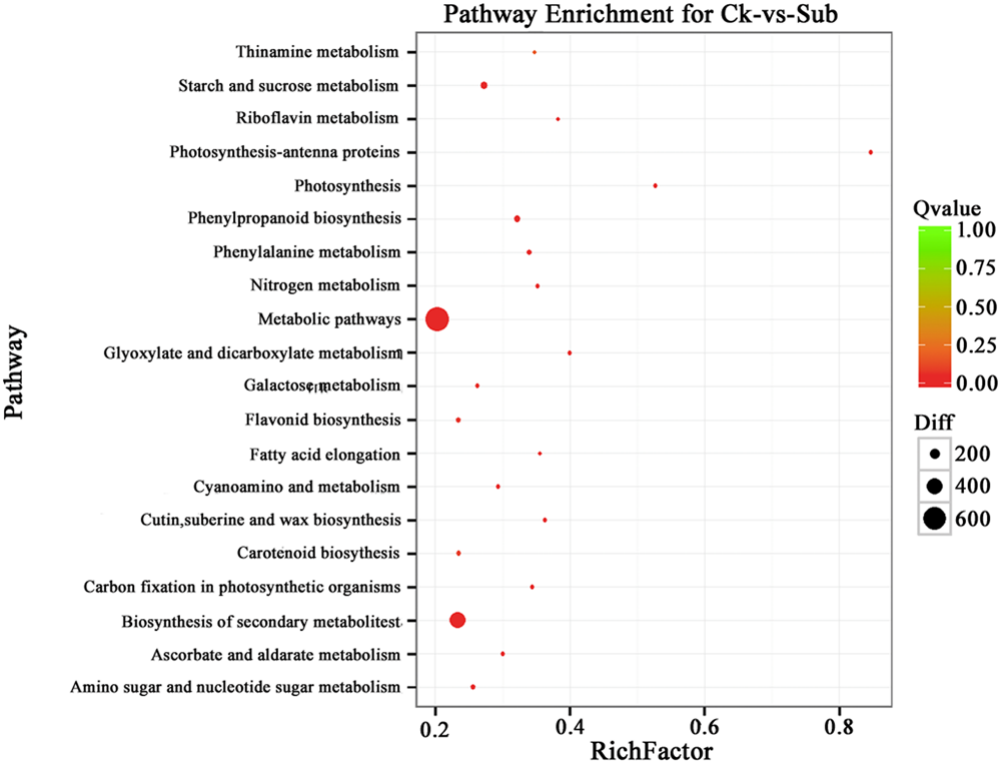
KEGG pathway enrichment of differentially expressed genes (DEGs). The left Y-axis shows the KEGG pathway name. The X-axis shows the enrichment factor. A high q-value is represented by green and a low q-value is represented by red (q < 0.05). Bubble size indicates DEG number (increases with DEG number).

The Kyoto encyclopedia of genes and genomes (KEGG) enrichment analysis was also performed and depicted in Fig. 5. Among DEGs of the two groups, two pathways including metabolic pathways, and biosynthesis of secondary metabolites (q < 0.05; Fig. 5) were most affected by submergence. Under complete submergence conditions, the decreased gas exchange leads to hypoxic conditions. Previous reports on some submergence resistant plants showed that available energy under submergence is largely obtained from anaerobic metabolic pathways, such as glycolysis, ethanol fermentation, pentose phosphate pathway, nitrate reduction, and cytoplasmic maintenance, etc. Thus, the biological processes identified here might provide plants the necessary energy in hypoxic environments to maintain normal physiological functions and alter morphology and growth upon submergence^26,27^.

### Molecular response of lotus upon complete submergence

**Figure 6:**
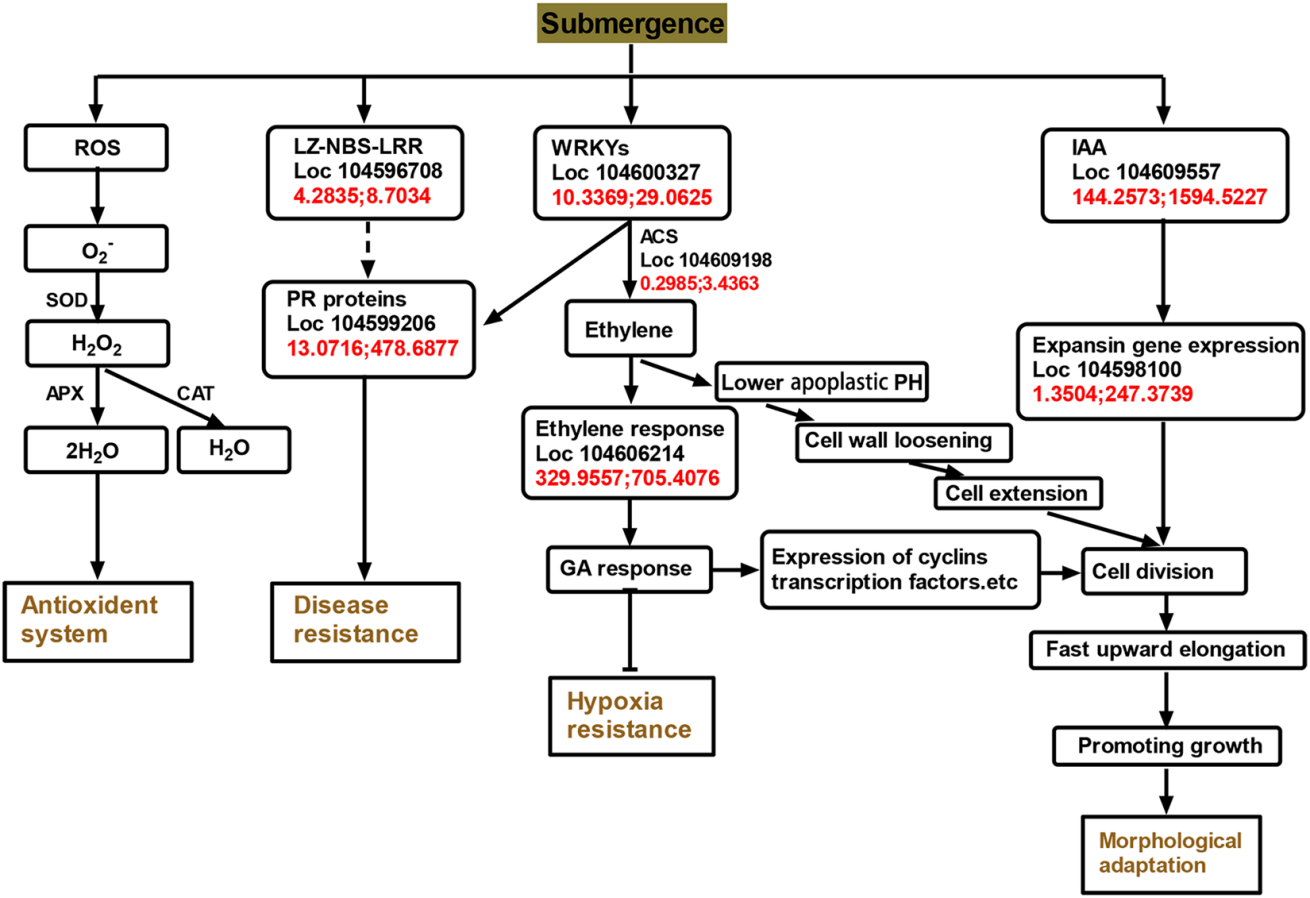
Model of possible regulatory network in lotus upon submergence stress. Based on regulatory networks, functional annotations and literature mining, a potential regulatory network of submergence-responsive genes in lotus was constructed. The model shows antioxidant system, disease response, hypoxia response, and morphological adaption contributing to defense response of lotus upon submergence.

Transcriptomic studies have revealed that expression of huge numbers of genes (10% of genes assayed) are altered upon flooding, suggesting that the transcriptional regulatory network plays important roles in regulating submergence responses in plants. Of the substantial number of genes identified as responding rapidly to submergence, we have chosen to focus on the subset exhibiting the greatest response to the submergence stress. Based on functional annotation and homologous alignments of DEGs to key genes in related models from previous works, a submergence stress-response regulatory network was proposed in lotus (Fig. 6). Reverse transcription quantitative PCR (RT-qPCR) analysis was conducted on some important genes in the model to verify the transcriptome data (Fig. S1). As expected, the changes in gene expression of 10 selected genes obtained from RT-qPCR was similar in magnitude to deep sequencing results (Fig. S1).

As shown in Fig. 6, we find some experimental evidence that homologous DEGs participated in two biological processes including stress tolerance and morphological adaptation. Under submergence stress, oxidative stress and ROS would be induced, which could generate lipid peroxidation and cause serious damages to biomembranes in plant cells. At the same time, some metabolites and enzymes including superoxide dismutase (SOD), peroxidase (POD), ascorbate peroxidase (APX), and catalase (CAT) were activated to decrease oxidative damage^28^. We also obtained some evidence that lotus produces more antioxidant enzymes and non-enzymic antioxidants upon submergence, which would alleviate submergence stress in plant cells. For example, genes encoding POD including Loc104597208 (POD, log_2_Sub/Ck = 2.45), Loc104593725 (POD5, log_2_Sub/Ck = 4.75), Loc104602472 (POD2, log_2_Sub/Ck = 2.49), Loc104589739 (POD-like, log_2_Sub/Ck = 4.32), Loc104600008 (POD3, log_2_Sub/Ck = 4.20), and Loc104588433 (POD, log_2_Sub/Ck = 6.35) increased over 2-fold. As a protective enzyme of organisms, POD synergizes with SOD, catalase and glutathione peroxidase to eliminate free radicals produced in plants under adverse conditions^29^. Upon submergence, the expression levels of the two glutathione S-transferase genes, LOC104598999 and LOC104594705, were increased under submergence stress. Glutathione transferase is a key enzyme in the glutathione binding reaction and catalyzes the initial step of the glutathione binding reaction^30^.

Elongation of submerged petioles allows leaves to emerge from water and thus restoring contact with the atmosphere which is an important mechanism of lotus to escape from submergence stress^31^. Previous studies have shown that ethylene-promoted underwater extension is one of the most compelling examples of hormone-mediated stress adaptation by plants^32^. Our previous work revealed that ethylene, abscisic acid(ABA), and gibberellin(GA) were all involved in regulating petiole elongation under submergence^33^. Upon submergence, increased ethylene played a critical role in modulating the balance between ABA (growth inhibiting) and GA (growth promoting) hormones and resulted in petiole elongation. GA action appears to be responsible for some of the cellular events linked to sustained fast cell elongation and division. In this work, we found several genes that encoded important components in phytohormone regulated networks were significantly differentially expressed when under submergence stress. For example, the expression of *ACS* gene, Loc104609198 was increased by 3.53-fold. Accordingly, we also found an ethylene response gene (Loc104606214) increased by over 1-fold after submergence treatment. In addition, IAA was also involved in accelerated growth of plants and enhanced the morphological adaptability of plants under flooding conditions^34^. We identified an IAA synthesis-related gene (Loc104609557, log_2_Sub/Ck = 3.47) and an expansin protein gene (Loc104598100, log_2_Sub/Ck = 7.52) that were significantly increased in response to submergence.

**Figure 7:**
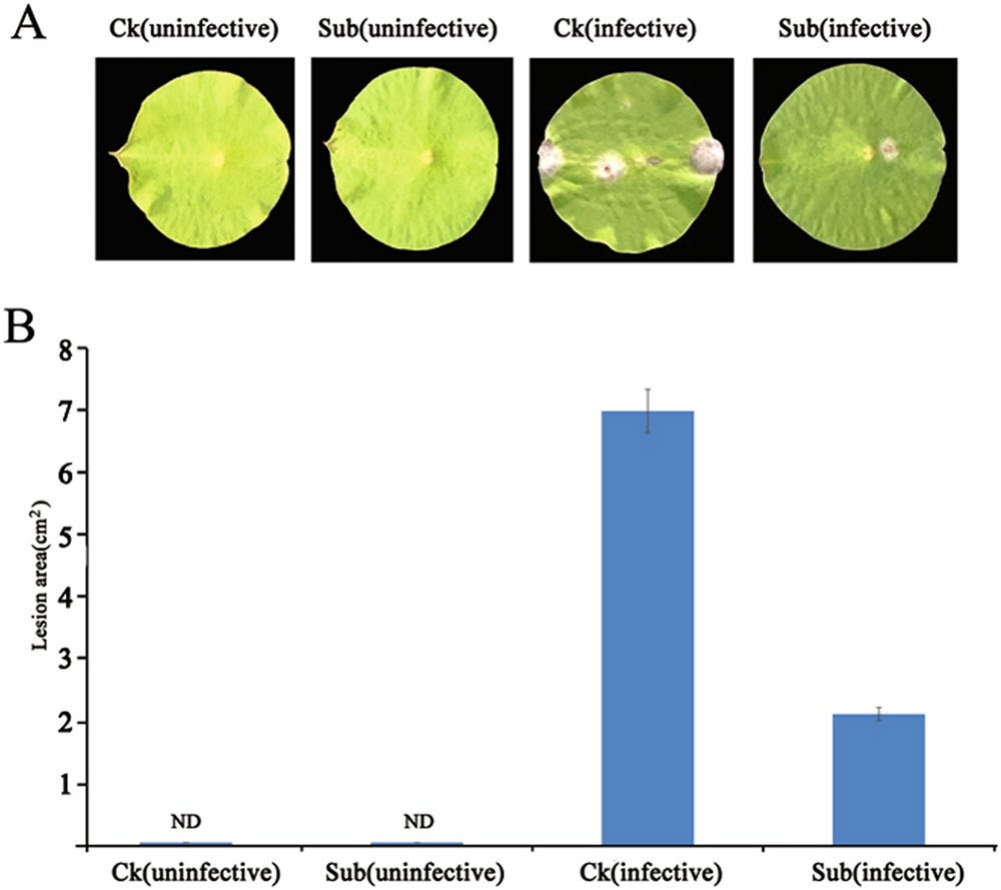
Submergence confers immunity to rot disease in lotus. (**A**) Disease symptoms assessed 3d postinoculation with *Fusarium oxysporum* in seedlings with or without submergence pretreatment. The data represented are from 12 independent repeats. (**B**) Statistical differences in lesion area between Ck and Sub. ND, not detected.

Besides antioxidant system and morphological responses, it is interesting to find that many WRKYs and innate immunity related genes are strongly induced during submergence (Fig. 6 and Table S2). We list only some of these genes that showed greatest response to the submergence (Table S2). This indicates that innate immunity of lotus may be induced during submergence. In natural conditions, heavy rain-induced submergence often causes tissue rotting, and results in a higher probability of pathogen infection^35^. Thus, after sensing the submergence signal, activating a suite of disease defense responses genes could effectively protect lotus from attack by microbial pathogens. Several lines of evidence add support to this conclusion. First, the expression of some *NBS-LRR* genes (the most induced gene: Loc104596708) were up-regulated under submergence, which might induce the expression of pathogenesis-related defense gene (PR) and improve its resistance to disease (Fig. 6 and Table S2)^36^. Second, some plant defense-related WRKY genes were strongly induced during submergence. For example, Loc104600327 in lotus is a homolog of grape WRKY30^37^, which was reported to enhanced grape resistance to downy mildew pathogen *Peronospora parasitica* mediated by increasing expression of many PR. Third, submergence pretreatment could enhance lotus immunity to rot disease. To determine whether lotus immunity was induced, we first submerged lotus seedlings and then assayed their immunity through inoculation with the rot disease pathogen *Fusarium oxysporum f*. *sp*. *Nelumbicola* (Fig. 7). After 3 days of infection, we observed that the lesion area of the Sub (pathogen infected) was significantly less than that of the Ck (pathogen infected) (Fig. 7A). Among the four most induced *WRKY* genes, we found that Loc104600327 (WRKY41 in lotus) were co-expressed with innate immunity marker genes following submergence, heat, shade, and copper stresses through WGCNA analysis of available transcriptome data from lotus, which indicated that Loc104600327 might mediate immunity in response to pathogen infection.

## Conclusion

In this work, we report the first comprehensive transcriptome-based characterization of submergence responsive genes in lotus. Expression of 1976 genes was up-regulated, and 2026 genes were down-regulated in lotus under submergence stress. Most of the DEGs were related to antioxidant system, disease resistance, hypoxia resistance and morphological adaptation. In addition, a model of a submergence stress-response regulatory network in lotus was proposed. These results may prove useful for better understanding the genetic mechanisms underlying disease resistance in lotus.

## Methods

### Plant materials, growth conditions and submergence treatment

Lotus (*Nelumbo nucifera* Gaertn) used in this study was grown under natural light in a greenhouse from May to August (Nanjing, China) with a temperature range of 13–35 °C. For submergence treatment, 3- month-old lotus seedlings were transferred into a plastic tank and water level was kept 20 cm above the top of seedlings. Seedlings grown simultaneously without submergence treatment served as the controls. After 12 h of submergence treatment, total RNA was extracted from whole seedlings with Trizol reagent (Invitrogen, USA) according to the instructions of the manufacturer.

### Inoculation method

Seeds of cultivar ‘Weishan Lake Honglian’ were germinated and seedlings were used as the plant host. Select strains of *Fusarium oxysporum* were used as the inoculum. The average size of the leaves was about 5 cm in diameter at inoculation. Due to the special structure of the lotus leaves, the upper surface of the leaves is waxy and hydrophobic, so syringes were used to suck the spore suspension of *Fusarium oxysporum* into the leaft to ensure that the bacteria can invade into the interior of the leaves. The untreated and treated leaves were cultured on agar medium and the changes in lesion area of the leaves infected by different strains were observed. The lesion area was measured with ImageJ software (version 1.48).

### RNA-seq library preparation and sequencing

For RNA-seq, three independent experiments with at least three replicates each were conducted and seedlings were sampled after control or submergence treatment. Library preparation and sequencing were performed with Illumina sequencing technology at the Total Genomics Solution (TGS) company (Shenzhen, China). Sequence data have been deposited with the GenBank data libraries under accession numbers PRJNA354065.

### Functional annotation

Function annotation was performed using a BLASTX program against the NCBI non-redundant (nr) protein database and the KEGG with a typical E value cut-off of 1e-5 to obtain reliable annotations. Protein genes that were significantly changed upon submergence treatment were categorized into cellular component, molecular function and biological process using Blast2GO program. Then, the web tool WEGO (http://wego.genomics.org.cn/) was used to plot GO annotations^38^. Morevoer, KEGG-based functional analysis was also performed to identify differentially expressed genes (DEG) involved in biological pathways.

### Identification and analysis of differentially expressed genes (DEGs)

Raw reads of both libraries were first filtered with NGS QC Toolkit (v2.3.3)^39^ to get clean reads. The lastest lotus genome sequence (deposited in NCBI) was used to build the mapping index using Bowtie2 (v2.1.0)^40^ and clean reads were then aligned to this indexed lotus genome using TopHat (v2.1.1)^41^. The relative abundances and differences between treatments were calculated using Cufflinks (v2.2.1) with default settings.

### RT-qPCR Validation

For gene expression analysis, total RNA were reverse transcribed to synthesize first-strand cDNA by One Step PrimeScript miRNA cDNA Synthesis Kit (TaKaRa) and the cDNA was used for RT-qPCR analysis. *Actin* gene was used as an internal control of RT-qPCR. RT-qPCR reactions were performed using a Mastercycler ep realplex real-time PCR system (Eppendorf, Hamburg, Germany) with SYBR Premix Ex Taq (TaKaRa) according to the manufacturer’s instructions. The relative expression level was presented as values relative to corresponding control samples after normalization. The specific primers were listed in Table S3.

## Electronic supplementary material

supplementary information
