# Supplementary material for: Genome-wide transcriptional analysis of submerged lotus reveals cooperative regulation and gene responses
Source: Sci Rep. 2018 Jun 15;8:9187. doi: 10.1038/s41598-018-27530-x (PMC6003939; doi:10.1038/s41598-018-27530-x)
Supplement: Supplementary file 1 — supplementary information [file 41598_2018_27530_MOESM1_ESM.docx]

**Manuscript title:**

Genome-wide transcriptional analysis of submerged lotus reveals cooperative regulation and gene responses

**Author list:**

Bei Wang, Qijiang Jin, Xiao Zhang, Neil S. Mattson**,** Huihui Ren, Jing Cao, Yanjie Wang, Dongrui Yao**,** Yingchun Xu

**Supplementary table:**

**Table S1** Number of reads sequenced and mapped to the lotus genome.

| Sample | Total reads | Total mapped reads | Unique mapped reads | Reads mapped in paired |
| --- | --- | --- | --- | --- |
| Sub | 50805686 | 78.89% | 75.00% | 70.40% |
| Ck | 50242328 | 88.19% | 86.66% | 82.90% |

**Table S2.** Differentially expressed genes under submergence stress.

| **GeneID** | **Ck** | **Sub** | **log2Fold** | **pvalue** | **qvalue** | **Diff** | **Description** |
| --- | --- | --- | --- | --- | --- | --- | --- |
| LOC104593725 | 316.0935 | 6396.134 | 4.748230576 | 0 | 0 | Up | PREDICTED: peroxidase 5-like [Nelumbo nucifera] |
| LOC104602472 | 2033.222 | 8605.038 | 2.490867503 | 0 | 0 | Up | PREDICTED: peroxidase 2-like [Nelumbo nucifera] |
| LOC104600008 | 184.6487 | 2553.803 | 4.199245916 | 0 | 0 | Up | PREDICTED: peroxidase 3-like [Nelumbo nucifera] |
| LOC104588433 | 34.42603 | 2112.022 | 6.348434919 | 0 | 0 | Up | peroxidase, putative [Ricinus communis] |
| LOC104597209 | 2397.304 | 5867.16 | 1.700703154 | 0 | 0 | Up | PREDICTED: cationic peroxidase 1-like [Nelumbo nucifera] |
| LOC104608049 | 5263.01 | 9293.868 | 1.229844705 | 0 | 0 | Up | PREDICTED: peroxidase 73-like [Nelumbo nucifera] |
| LOC104589740 | 488.2237 | 2388.136 | 2.699724449 | 0 | 0 | Up | bacterial-induced peroxidase precursor [Gossypium hirsutum] |
| LOC104604900 | 10.43213 | 984.3187 | 6.969473531 | 1.02E-261 | 4.88E-260 | Up | PREDICTED: peroxidase 27-like [Nelumbo nucifera] |
| LOC104602004 | 50.07423 | 698.5175 | 4.21160971 | 4.50E-177 | 1.45E-175 | Up | PREDICTED: cationic peroxidase 1-like [Nelumbo nucifera] |
| LOC104597565 | 30.25318 | 365.2443 | 4.003156743 | 6.84E-91 | 1.30E-89 | Up | Peroxidase 2 [Theobroma cacao] |
| LOC104597207 | 75.11134 | 434.9991 | 2.943363461 | 1.25E-84 | 2.26E-83 | Up | PREDICTED: peroxidase 4-like [Nelumbo nucifera] |
| LOC104587423 | 11.47534 | 223.7969 | 4.695034934 | 6.66E-61 | 9.02E-60 | Up | PREDICTED: peroxidase 3-like [Nelumbo nucifera] |
| LOC104602474 | 23.9939 | 235.4227 | 3.703967542 | 2.35E-56 | 3.00E-55 | Up | PREDICTED: cationic peroxidase 1-like [Nelumbo nucifera] |
| LOC104585679 | 0.001 | 103.6635 | 8.105217886 | 8.86E-27 | 6.49E-26 | Up | PREDICTED: peroxidase 27-like [Nelumbo nucifera] |
| LOC104592873 | 16.69141 | 77.50541 | 2.624644872 | 8.92E-15 | 4.28E-14 | Up | PREDICTED: peroxidase 51 [Nelumbo nucifera] |
| LOC104602473 | 9.388917 | 54.25379 | 2.94014695 | 5.96E-12 | 2.52E-11 | Up | PREDICTED: peroxidase P7-like [Nelumbo nucifera] |
| LOC104599982 | 1.043213 | 24.22044 | 4.946573102 | 4.68E-08 | 1.54E-07 | Up | PREDICTED: cationic peroxidase 1-like isoform X2 [Nelumbo nucifera] |
| LOC104585678 | 0.001 | 20.34517 | 5.756068117 | 4.11E-07 | 1.26E-06 | Up | PREDICTED: peroxidase 27-like [Nelumbo nucifera] |
| LOC104604497 | 25.03711 | 59.09788 | 1.648492021 | 4.74E-07 | 1.45E-06 | Up | PREDICTED: peroxidase 17-like [Nelumbo nucifera] |
| LOC104610571 | 6.259278 | 31.00216 | 2.717754263 | 5.83E-07 | 1.76E-06 | Up | PREDICTED: peroxidase 5-like [Nelumbo nucifera] |
| LOC104605302 | 728 | 1183 | 1.003156284 | 1.78E-51 | 2.11E-50 | Up | PREDICTED: probable WRKY transcription factor 34 isoform X2 [Nelumbo nucifera] |
| LOC104600327 | 379 | 864 | 1.491550636 | 1.64E-69 | 2.51E-68 | Up | Probable WRKY transcription factor 41 [Nelumbo nucifera (sacred lotus) ] |
| LOC104597162 | 43 | 156 | 2.161854864 | 4.32E-22 | 2.74E-21 | Up | probable WRKY transcription factor 49 [Nelumbo nucifera (sacred lotus) ] |
| LOC104586751 | 92 | 521 | 2.804294545 | 2.44E-93 | 4.78E-92 | Up | PREDICTED: probable WRKY transcription factor 72 [Nelumbo nucifera] |
| LOC104598999 | 1946.636 | 3412.176 | 1.219162608 | 3.5E-204 | 1.31E-202 | Up | glutathione S-transferase [Vitis vinifera] |
| LOC104594705 | 55.29029 | 116.2581 | 1.481686878 | 6.35E-11 | 2.53E-10 | Up | PREDICTED:glutathione S-transferase L3-like isoform X2 [Nelumbo nucifera] |
| LOC104606659 | 21.90747 | 174.3872 | 3.402253048 | 1.21E-39 | 1.17E-38 | Up | putative pathogenesis related protein 1 precursor [Vitis vinifera] |
| LOC104599206 | 288.97 | 7968.525 | 5.194774758 | 0.00E+00 | 0.00E+00 | Up | pathogenesis-related thaumatin-like protein [Ziziphus jujuba] |
| LOC104588992 | 640.5328 | 4854.745 | 3.331504851 | 0.00E+00 | 0.00E+00 | Up | PREDICTED: pathogenesis-related protein STH-2-like [Nelumbo nucifera] |
| LOC104590419 | 501.7855 | 2434.639 | 2.688018869 | 0.00E+00 | 0.00E+00 | Up | pathogenesis-related (PR)-10-related norcoclaurine synthase-like protein [Eschscholzia californica] |
| LOC104588993 | 1084.942 | 3044.025 | 1.897815974 | 0.00E+00 | 0.00E+00 | Up | Pathogenesis-related protein 10.9 [Theobroma cacao] |
| LOC104593969 | 9.388917 | 892.2811 | 6.979849403 | 2.68E-237 | 1.15E-235 | Up | pathogenesis-related protein PR-1 precursor [Capsicum annuum] |
| LOC104593441 | 721.9034 | 1595.643 | 1.55371389 | 9.50E-139 | 2.54E-137 | Up | pathogenesis-related family protein [Populus trichocarpa] |
| LOC104612628 | 139.7905 | 597.7605 | 2.505754933 | 8.78E-98 | 1.78E-96 | Up | PREDICTED: pathogenesis-related protein 1-like [Nelumbo nucifera] |
| LOC104593971 | 6.259278 | 187.9506 | 5.317667143 | 4.07E-53 | 4.97E-52 | Up | pathogenesis-related protein PR-1 precursor [Capsicum annuum] |
| LOC104588365 | 508.0447 | 865.1542 | 1.177455542 | 1.97E-50 | 2.30E-49 | Up | PREDICTED: pathogenesis-related genes transcriptional activator PTI6-like [Nelumbo nucifera] |
| LOC104595510 | 1.043213 | 28.09571 | 5.160697887 | 3.32E-09 | 1.19E-08 | Up | PREDICTED: probable indole-3-acetic acid-amido synthetase GH3.5 [Nelumbo nucifera] |
| LOC104587077 | 3.129639 | 31.97098 | 3.762148495 | 4.59E-09 | 1.63E-08 | Up | PREDICTED: indole-3-acetic acid-induced protein ARG7-like [Nelumbo nucifera] |
| LOC104604362 | 2.086426 | 24.22044 | 3.946573102 | 2.18E-07 | 6.84E-07 | Up | PREDICTED: indole-3-acetic acid-induced protein ARG7-like [Nelumbo nucifera] |
| LOC104587078 | 0.001 | 17.43872 | 5.533675932 | 2.81E-06 | 7.97E-06 | Up | PREDICTED: indole-3-acetic acid-induced protein ARG7-like [Nelumbo nucifera] |
| LOC104603960 | 2185.531 | 3465.461 | 1.074517127 | 1.02E-169 | 3.14E-168 | Up | Indole-3-acetic acid-amido synthetase GH3.5, putative isoform 1 [Theobroma cacao] |
| LOC104609557 | 3553.184 | 29574.13 | 3.46660511 | 0 | 0 | Up | PREDICTED: auxin-responsive protein IAA16-like [Nelumbo nucifera] |
| LOC104595142 | 1739.036 | 4128.132 | 1.656654991 | 0 | 0 | Up | PREDICTED: auxin-responsive protein IAA27-like [Nelumbo nucifera] |
| LOC104609555 | 197.1673 | 848.6843 | 2.515261373 | 1.37E-138 | 3.66E-137 | Up | AUX/IAA transcriptional regulator family protein isoform 1 [Theobroma cacao] |
| LOC104612448 | 1395.819 | 2319.349 | 1.142061735 | 7.18E-126 | 1.79E-124 | Up | AUX/IAA transcriptional regulator family protein [Theobroma cacao] |
| LOC104595708 | 14.60498 | 281.9259 | 4.68023742 | 3.74E-76 | 6.24E-75 | Up | PREDICTED: IAA-amino acid hydrolase ILR1-like 1 [Nelumbo nucifera] |
| LOC104586117 | 131.4448 | 311.9593 | 1.656354436 | 3.64E-31 | 2.98E-30 | Up | PREDICTED: auxin-responsive protein IAA28-like [Nelumbo nucifera] |
| LOC104600214 | 9.388917 | 27.12689 | 1.940146684 | 0.000134575 | 0.000320785 | Up | 1-aminocyclopropane-1-carboxylate synthase [Eriobotrya japonica] |
| LOC104609198 | 9.388917 | 81.38068 | 3.525109362 | 6.88E-20 | 4.05E-19 | Up | PREDICTED: 1-aminocyclopropane-1-carboxylate synthase 3-like [Nelumbo nucifera] |

**Table S3** Primers used for RT-qPCR.

| Gene | Forward primer (5’-3’) | Reverse primer (5’-3’) |
| --- | --- | --- |
| Loc104600327 | TCACCTACAGAGGAAAGCACA | CTTGAGTCCCGTCCGAAA |
| Loc104609198 | CGAGTGGGTGCAATTTACTCTA | AGCCTCCTCTGGTTCTCTATAA |
| Loc104609557 | GGAAAATGGCGAGCAGG | CAACAGTCTCAGCGAACCC |
| Loc104605302 | GACTGTTCTCGGCTTTCATTT | GCATTAACGCCAGACCCA |
| Loc104597162 | GAGGAAGTATGGGCAGAAATC | GGAGGGAAGGAAATGTGAGT |
| Loc104586751 | CCGAAATGGGTGAAGTGAG | TTGGTCTTTAGGGGCTGTATC |
| Loc104598100 | ACGGTGGAGGGTGGATTA | GTGGGCAGAAGTTAGTGGC |
| Loc104599206 | GTTTGTCCAGTGGTGGGTTG | TGAGGGAAGGGCTATCGTG |
| Loc104596708 | CAGCAACTGAGACGAACCTACT | CCAGACCGCCCATACCA |
| Loc104606214 | CCCTCGCAATAGAAACCG | CACCAGCAGCACCGTCA |

**Supplementary figure:**

**Fig. S1** quantitative RT-PCR method to verify the differentially expressed genes.

**Fig. S1**

**
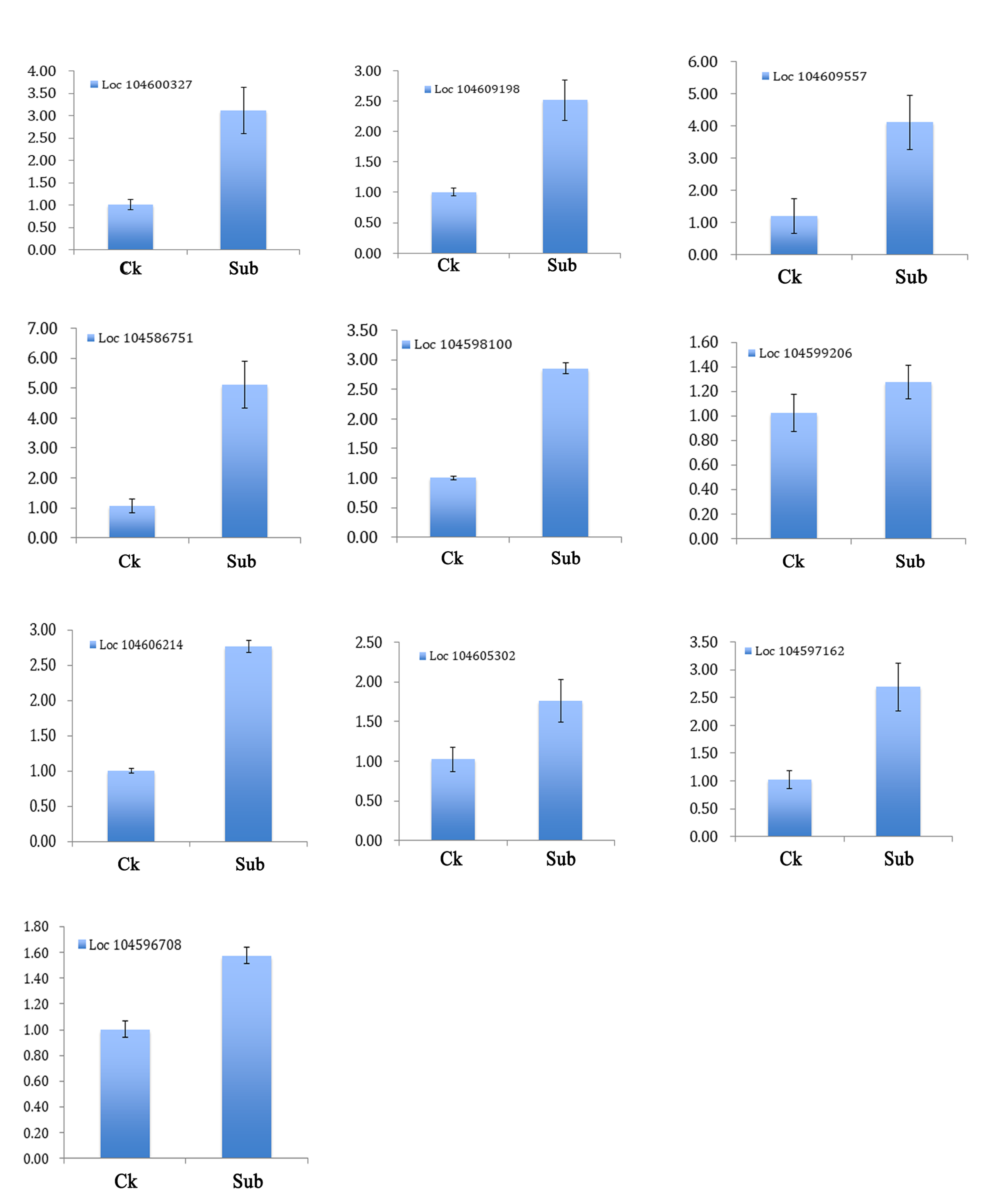
**
